# Intracellular activity and *in vivo* efficacy in a mouse model of septic arthritis of the novel pseudopeptide Pep16 against *Staphylococcus aureus* clinical isolates

**DOI:** 10.1093/jacamr/dlae025

**Published:** 2024-02-26

**Authors:** Jean-Baptiste Mascary, Valérie Bordeau, Irène Nicolas, Marie-Clémence Verdier, Pierre Rocheteau, Vincent Cattoir

**Affiliations:** Inserm U1230 BRM (Bacterial RNAs and Medicine), Université de Rennes, Rennes, France; SAS Olgram, Bréhan, France; Inserm U1230 BRM (Bacterial RNAs and Medicine), Université de Rennes, Rennes, France; SAS Olgram, Bréhan, France; CHU de Rennes, Service de Pharmacologie, Rennes, France; SAS Olgram, Bréhan, France; CHU de Rennes, Service de Bactériologie-Hygiène hospitalière, 2 rue Henri Le Guilloux, 35033 Rennes, France; CNR de la Résistance aux Antibiotiques (laboratoire associé ‘Entérocoques’), CHU de Rennes, Rennes, France

## Abstract

**Objectives:**

Assessing the therapeutic potential of a novel antimicrobial pseudopeptide, Pep16, both *in vitro* and *in vivo* for the treatment of septic arthritis caused by *Staphylococcus aureus*.

**Methods:**

Seven clinical isolates of *S. aureus* (two MRSA and five MSSA) were studied. MICs of Pep16 and comparators (vancomycin, teicoplanin, daptomycin and levofloxacin) were determined through the broth microdilution method. The intracellular activity of Pep16 and levofloxacin was assessed in two models of infection using non-professional (osteoblasts MG-63) or professional (macrophages THP-1) phagocytic cells. A mouse model of septic arthritis was used to evaluate the *in vivo* efficacy of Pep16 and vancomycin. A preliminary pharmacokinetic (PK) analysis was performed by measuring plasma concentrations using LC-MS/MS following a single subcutaneous injection of Pep16 (10 mg/kg).

**Results:**

MICs of Pep16 were consistently at 8 mg/L for all clinical isolates of *S. aureus* (2- to 32-fold higher to those of comparators) while MBC/MIC ratios confirmed its bactericidal activity. Both Pep16 and levofloxacin (when used at 2 × MIC) significantly reduced the bacterial load of all tested isolates (two MSSA and two MRSA) within both osteoblasts and macrophages. In MSSA-infected mice, Pep16 demonstrated a significant (∼10-fold) reduction on bacterial loads in knee joints. PK analysis following a single subcutaneous administration of Pep16 revealed a gradual increase in plasma concentrations, reaching a peak of 5.6 mg/L at 12 h.

**Conclusions:**

Pep16 is a promising option for the treatment of septic arthritis due to *S. aureus*, particularly owing to its robust intracellular activity.

## Introduction

*Staphylococcus aureus* remains a problematic pathogen implicated in a spectrum of bone and joint infections (BJIs), which encompass septic arthritis, acute and chronic osteomyelitis and spondylodiscitis, as well as infections related to orthopaedic devices.^1–3^ Septic arthritis, in particular, stands out due to its rapid progression and the extensive damage it inflicts on joints and cartilage. Collectively, BJIs can lead to long-lasting disability and place a substantial burden on healthcare systems.^4^

Although traditional antibiotics have been employed in the fight against *S. aureus*, the emergence of antibiotic resistance, particularly with the widespread prevalence of MRSA isolates, presents a substantial challenge to achieving effective treatment outcomes.^5^ The growing complexity of treating BJIs arises not only from the ability of *S. aureus* to invade and persist within professional (e.g. macrophages) and non-professional (e.g. osteoblasts) phagocytic cells^6^ but also from its capacity to form biofilms.^7^ These intricacies significantly compound the challenges of treatment.^8–12^ Consequently, there is an urgent need to explore innovative antibacterial strategies capable of effectively addressing both intracellular and biofilm-associated forms of *S. aureus*.

Among the emerging alternatives, antimicrobial peptides (AMPs) have become promising therapeutic options due to their established ability to modulate symbiotic bacterial populations.^13,14^ These small peptides are ubiquitous across all domains of life and play an integral role in an organism immune system.^15^ They typically possess broad-spectrum antimicrobial activity against bacteria, viruses and fungi.^16^ Recent advancements have led to the engineering of pseudomimetic AMPs, derived from a toxin naturally produced by *S. aureus*.^17^ These pseudopeptides exhibit bactericidal properties against a range of MDR Gram-negative and Gram-positive pathogens, including MRSA.^18^ While their exact mechanism of action remains unclear, they appear to primarily disrupt the integrity of bacterial cell membranes, leading to bacteriolysis.^18^ One such peptide of considerable interest is Pep16, which has exhibited promising outcomes in mouse sepsis models caused by MRSA.^18^ Importantly, Pep16 administration has shown minimal toxicity and a reduced likelihood of bacterial resistance emergence, positioning it as a favourable candidate for antimicrobial drug development.^18^ Given the significant burden posed by BJIs, particularly septic arthritis caused by *S. aureus*, we explored the potential of Pep16 as a viable treatment option for these infections.

In this study, our objective was to assess the efficacy of Pep16, both *in vitro* and *in vivo*, for eradicating *S. aureus* in BJIs. Firstly, we evaluated the *in vitro* activity of Pep16, either alone or in combination with other agents, comparing it with commonly used antibiotics for the BJI treatment. Secondly, we determined the intracellular activity of Pep16 using two relevant cell-based infection models involving osteoblasts and macrophages. Finally, we assessed the *in vivo* efficacy of Pep16 using a mouse model of septic arthritis.

## Materials and methods

### Bacterial strains and antimicrobial susceptibility testing (AST)

A total of seven clinical isolates of *S. aureus* obtained from patients suffering from septic arthritis in 2020–21 were studied, as detailed in Table 1. Additionally, the MSSA reference strain ATCC 29213 was used as control for AST.

**Table 1.** *In vitro* activity of Pep16 and comparators against the seven clinical isolates of *S. aureus* responsible for septic arthritis

*S. aureus* strain	Origin (location, date)	Resistance phenotype	ST	*spa* type	Resistance mechanisms	Virulence genes	MIC/MBC (mg/L)				
							Pep16	VAN	TEC	DAP	LVX
1333	Clinical, joint fluid (Rennes, September 2020)	MSSA, multi^S^	398	t34	*blaZ*	*aur*, *hlgA*, *hlgB*, *hlgC*	8/16	0.5/0.5	0.5/1	0.25/0.5	0.5/0.5
1334	Clinical, joint fluid (Rennes, November 2020)	MSSA, multi^S^	New^a^	t548	—	*aur*, *splA*, *splB*, *hlgA*, *hlgB*, *hlgC*, *lukD*, *lukE*, *seg*, *sei*, *sem*, *sen*, *seo*, *seu*, *sak*, *scn*	8/16	0.5/0.5	0.25/0.5	0.25/0.5	0.5/0.5
1814	Clinical, joint fluid (Rennes, September 2020)	MRSA, cip^R^	30	t363	*blaZ*, *mecA*, *dfrG* Mutations in *gyrA* (S84L) and *parC* (S80Y)	*aur*, *splE*, *hlgA*, *hlgB*, *hlgC*, *lukF-PV*, *lukS-PV*, *seg*, *sei*, *sem*, *sen*, *seo*, *seu*, *sak*, *scn*	8/16	0.5/0.5	0.5/1	0.25/0.5	4/4
1815	Clinical, joint fluid (Rennes, March 2021)	MRSA, gen^R^, ery^R^	8	t1476	*blaZ*, *mecA*, *aac(6′)-aph(2′′)*, *erm*(C), *dfrG* Mutation in *parC* (S80Y)	*aur*, *splA*, *splB*, *splE*, *hlgA*, *hlgB*, *hlgC*, *lukD*, *lukE*, *tst*, *sak*, *scn*	8/16	0.5/0.5	0.5/1	0.25/0.5	0.5/0.5
1816	Clinical, joint fluid (Rennes, January 2021)	MSSA, multi^S^	398	t6608	—	*aur*, *hlgA*, *hlgB*, *hlgC*, *scn*	8/16	0.5/0.5	0.25/0.5	0.25/0.5	0.25/0.25
1817	Clinical, joint fluid (Rennes, February 2021)	MSSA, ery^R^	398	t571	*blaZ*, *erm*(T)	*aur*, *hlgA*, *hlgB*, *hlgC*, *scn*	8/16	0.5/0.5	0.5/1	0.25/0.5	0.25/0.25
1818	Clinical, joint fluid (Rennes, February 2021)	MSSA, multi^S^	12	t156	—	*aur*, *splA*, *splB*, *splE*, *hlgA*, *hlgB*, *hlgC*, *lukD*, *lukE*, *sec*, *sel*, *sep*, *sak*, *scn*	8/16	0.5/0.5	1/2	0.25/0.5	0.25/0.25
ATCC 29213	Reference	MSSA, multi^S^	nd	nd	nd	nd	8/16	0.5/0.5	0.5/1	0.25/0.5	0.5/0.5

VAN, vancomycin; TEC, teicoplanin; DAP, daptomycin; LVX, levofloxacin; cip^R^, ciprofloxacin resistant; ery^R^, erythromycin resistant (inducible MLS_B_ phenotype); gen^R^, gentamicin resistant (KTG phenotype); multi^S^, multi-susceptible; nd, not determined.

^a^New ST close to ST2975.

AST was conducted through the disc diffusion method, following the guidelines outlined by Comité de l’Antibiogramme de la Société Française de Microbiologie (CA-SFM)/EUCAST (www.sfm-microbiologie.org/boutique/comite-de-lantibiograme-de-la-sfm-casfm/). MIC and MBC values of Pep16 (synthesized by Olgram, France), as well as those of vancomycin, teicoplanin, daptomycin and levofloxacin, were determined using the broth microdilution reference method.

The presence of *in vitro* synergy between Pep16 and clinically relevant antibiotics was assessed through chequerboard synergy assay. Combinations of Pep16 and various antibiotics (vancomycin, teicoplanin, daptomycin, levofloxacin or rifampicin) were tested at concentrations in the ranges 1–64 and 0.03–32 mg/L, respectively. The FIC index was calculated by summing the FICs of Pep16 and the respective antibiotic. Synergy was defined as FIC index < 0.5.

Time–kill curves were performed with Pep16 (80 or 160 mg/L) against four clinical isolates of *S. aureus* from patients suffering from septic arthritis. Bacterial cell suspensions grown overnight in CAMHB (CA-MHB II) and was diluted in pure CAMHB to obtain a suspension calibrated at 0.5 MacFarland, then diluted 1:5 in CAMHB to obtain a suspension of 2 × 10^7^ cfu/mL. One millilitre of this suspension was inoculated into 20 mL of antibiotic solution (expected final bacterial concentration of 10^6^ cfu/mL). Viable counts were enumerated by plating 100 μL of appropriate culture dilutions onto brain heart infusion (BHI) agar plates after 0, 3, 6, 8, 11 and 24 h of incubation at 37°C and expressed in log_10_ cfu/mL. A bactericidal effect was defined as a 3 log_10_ decrease in cfu counts compared with the initial inoculum.

### WGS and bioinformatic analysis

Genomic DNA was isolated using the Quick-DNA fungal/bacterial miniprep kit (Zymo Research, Irvine, CA, USA). DNA libraries were prepared using the NEBNext Ultra DNA library prep kit for Illumina (New England Biolabs, Ipswich, MA, USA). The libraries were then sequenced as paired-end reads (2 × 300 bp) using an Illumina MiSeq platform and the MiSeq reagent kit version 3. The Illumina reads were assembled using the SPAdes software and the annotation of chromosome and plasmids was performed using the NCBI Prokaryotic Genome Annotation Pipeline (PGAP) (www.ncbi.nlm.nih.gov/genome/annotation_prok/). MLST and staphylococcal protein A (*spa*) typing were performed using the MLST database version 2.0 (https://cge.food.dtu.dk/services/MLST/) and the spaTyper software version 1.0 (https://cge.food.dtu.dk/services/spaTyper/). The nucleotide sequences were also submitted to ResFinder 4.4.1 (http://genepi.food.dtu.dk/resfinder) and VirulenceFinder 2.0 (https://cge.food.dtu.dk/services/VirulenceFinder/) to identify antimicrobial resistance mechanisms and virulence genes, respectively. Genomic sequences of the seven strains were deposited in GenBank under project PRJNA1040494.

### Cellular-based models of infection

The osteoblast infection model was conducted using MG-63 (CRL-1427) osteoblastic cells, following previously published procedures.^19^ Briefly, MG-63 cells were cultured at 37°C in a 5% CO_2_ environment and passaged weekly for up to 20 weeks. The culture medium used was DMEM supplemented with 10% FBS, 25 mM HEPES, 2 mM L-glutamine, with or without antibiotics (100 U/mL penicillin plus 100 mg/L streptomycin). For the assays, MG-63 cells were seeded in 24-well plates until reaching 70%–80% confluence. Bacterial cell suspensions grown overnight in BHI broth were prepared and adjusted to an moi of 50, and then introduced to the osteoblasts. After a 2 h coincubation to facilitate bacterial adhesion, MG-63 cells were rinsed and treated with 100 mg/L gentamicin for 1 h to eliminate extracellular bacterial cells. Infected osteoblasts were subsequently exposed to Pep16 or levofloxacin (at 2 × MIC) for 3 h in the absence of FBS. Finally, intracellular bacterial cells were quantified in cellular lysates (following osmotic shock) by plating them onto BHI agar.

The macrophage infection model was performed using the THP-1 human monocytic cell line (ATCC, Rockville, USA). In brief, cells were cultured in the Roswell Park Memorial Institute (RPMI) medium supplemented with 10% FBS in T75 flasks at 37°C and 5% CO_2_, and were maintained through weekly transfers within passages 2 to 20. For the assays, THP-1 cells were seeded in 12-well plates and differentiated into macrophages with phorbol myristate acetate (PMA) for 72 h until they reached approximately 80% confluence. Bacterial suspensions, grown overnight in BHI, were prepared and adjusted to an moi of 10, and then introduced to the macrophages. After a 2 h coincubation to facilitate bacterial adherence, the cells were washed and treated with 100 mg/L gentamicin for 1 h. Infected macrophages were subsequently exposed to Pep16 or levofloxacin (at 2 × MIC) for 24 h in the absence of FBS. Finally, intracellular bacterial cells were enumerated from cellular lysates (following osmotic shock) by plating them onto BHI agar. Infected but untreated cells were used as a control in all experiments. The number of cfu in treated cells was standardized as a percentage of the controls.

In both models, the viability was assessed using the MTT assay to determine the potential cytotoxicity of antimicrobials on untreated and infected/treated cells. Briefly, an MTT solution (0.5 mg/mL) was added to the cell medium without FBS. After washing with PBS and a 30 min incubation at 37°C, the formazan crystals formed were dissolved in DMSO. Absorbance values at 540 nm were measured using a BioTek Synergy 2 microplate reader.

### Mouse model of septic arthritis

A mouse model of septic arthritis was employed to assess the *in vivo* effectiveness of Pep16 in comparison with vancomycin, following previously described procedures.^20^ Female Swiss mice, obtained from Janvier Labs and aged between 11 and 13 weeks with an approximate weight of 34 g, were used. The experiments were conducted in the ARCHE animal facility (Biosit) in Rennes. Mice were inoculated IV with 200 μL of a 0.9% NaCl solution containing approximately 10^8^ cfu of the 1334 strain (MSSA). Three hours after inoculation, infected mice received a subcutaneous dose of either a saline solution (0.9% NaCl), Pep16 (10 mg/kg) or vancomycin (100 mg/kg). These treatments were administered daily for a duration of 7 days. Each experimental group included a maximum of five mice and the experiments were repeated twice for the vancomycin group and three times for the 0.9% NaCl and Pep16 groups. The mice were monitored on a daily basis, and on the seventh day, they were humanely euthanized using CO_2_. Subsequently, knee joints, spleen and kidneys were excised, weighed and homogenized in 0.9% NaCl using a Polytron PT 3100D homogenizer (Kinematica, Switzerland). Bacterial loads were determined by plating serial dilutions onto BHI agar and expressed as log_10_ cfu/g.

### Pep16 pharmacokinetic analysis

Following subcutaneous injection of Pep16 (10 mg/kg), plasma concentrations were assessed at various timepoints including 0, 0.5, 1, 3, 6, 12, 15 and 24 h in non-infected mice. Blood samples were collected, subjected to centrifugation and the resulting plasma was stored at −80°C. Quantification was carried out utilizing a previously described LC-MS/MS method.^21^

### Statistical methods

For continuous variables, means and their standard deviations (SDs) were presented and compared using the Mann–Whitney *U*-test. Proportions were compared using a Fisher’s exact or chi-squared test, when appropriate. Statistical analyses were conducted using Prism software v9.0 (GraphPad, San Diego, CA, USA). A *P* value of <0.05 was considered statistically significant.

### Ethics

All experimental protocols were approved by the Adaptive Therapeutics Animal Care and Use Committee (APAFiS #4508-201603111554125_v5). The animals were housed in compliant cages and provided with free access to food and water.

## Results

### Characterization of clinical isolates

Out of the seven clinical isolates of *S. aureus*, two were MRSA (1814 and 1815) while the remaining five were MSSA (1333, 1334, 1816, 1817 and 1818) (Table 1). One MRSA isolate displayed ciprofloxacin resistance, while the other MRSA isolate exhibited resistance to gentamicin (KTG phenotype) and erythromycin (inducible MLS_B_ phenotype) but remained susceptible to fluoroquinolones. Among the five MSSA isolates, four displayed susceptibility to multiple antibiotics, with only one (1817) exhibiting a resistance trait (inducible MLS_B_ phenotype).

Thanks to WGS analysis, we determined that the seven isolates belonged to five STs (including three strains being ST398) and seven different spa types. As expected, both MRSA isolates harboured a *mecA* gene and the presence or absence of other resistance genes correlated with resistance phenotypes in all cases. Interestingly, several virulence genes were detected for all strains including exoenzymes, toxins and immunomodulatory proteins. Notably, the MRSA strain 1814 possessed *lukF-PV* and *lukS-PV* genes coding for Panton–Valentine leucocidin (PVL) while the MSSA strain was positive for the *tst* gene coding for toxic shock syndrome toxin-1 (TSST-1).

### In vitro activity of Pep16

Subsequently, we assessed the *in vitro* activity of Pep16 and compared it with commonly used antibiotics for treating BJIs caused by *S. aureus.* This assessment involved determining both MIC and MBC values. Notably, the MICs of Pep16 for all *S. aureus* isolates were consistently 8 mg/L. In contrast, the MICs for vancomycin, teicoplanin, daptomycin and levofloxacin were found to be 0.5, 0.25–1, 0.25 and 0.25–4 mg/L, respectively (Table 1). Although Pep16 exhibited MICs 2-to-32-fold higher than those of the comparator antibiotics, the MBC/MIC ratios indicated bactericidal activity, with ratios consistently at or below 4 for all strains (Table 1). This bactericidal activity was confirmed by establishing time–kill curves with concentrations of 10 × MIC and 20 × MIC (Figure S1, available as Supplementary data at *JAC-AMR* Online). Furthermore, we employed a chequerboard synergy assay to explore combinations of Pep16 with various clinically relevant antibiotics (vancomycin, teicoplanin, daptomycin, levofloxacin or rifampicin). Unfortunately, this analysis did not reveal any synergy (Figure S2).

### Intracellular activity of Pep16

We assessed the potential intracellular activity of Pep16 using two cellular infection models, one involving non-professional phagocytic cells (osteoblasts) and the other using professional phagocytic cells (macrophages), by comparison with levofloxacin.

**Figure 1.**
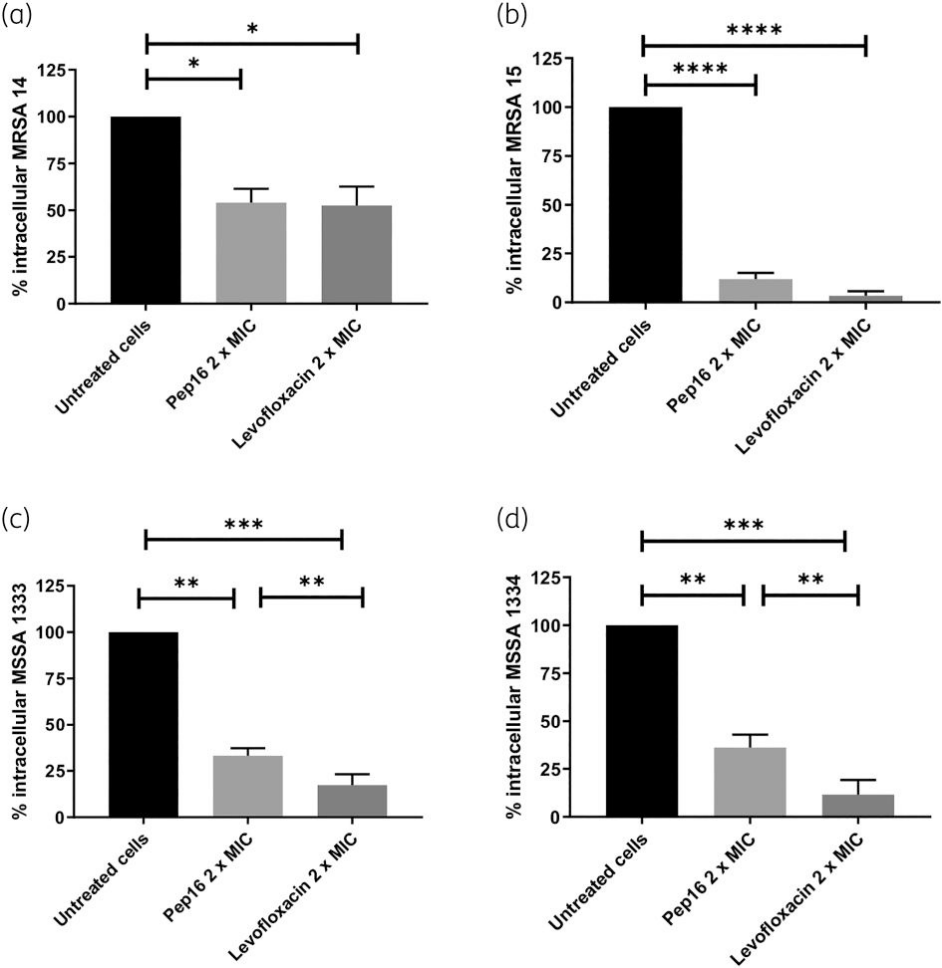
Intracellular activity of Pep16 and levofloxacin against intra-osteoblastic *S. aureus* MRSA 1814 (a), MRSA 1815 (b), MSSA 1333 (c) and MSSA 1334 (d). At 3 h post-infection, MG-63 cells were treated with Pep16 or levofloxacin at 2 × MIC for 3 h. Data represent the mean ± SD from three independent experiments. Intracellular cfu counts were normalized relative to untreated cells (depicted by black histograms), and differences between treated and untreated cells, as well as between the two compounds, were assessed using the Mann–Whitney *U*-test. *P < 0.05; **P < 0.01; ***P < 0.001; ****P < 0.0001.

In the acute model of osteoblast infection, we observed that both Pep16 and levofloxacin (administered at 2 × MIC) significantly reduced the bacterial load of all four tested *S. aureus* isolates (2 MSSA and 2 MRSA) 3 h after treatment, in comparison with untreated cells (Figure 1). Pep16 notably decreased the intracellular inoculum of both MRSA and MSSA, with reductions ranging from 46.5% to 86.6% for MRSA isolates and approximately 63% for MSSA isolates (Figure 1). Although levofloxacin appeared to be significantly more effective than Pep16 against MSSA isolates, no significant differences were observed between these compounds against intracellular MRSA (Figure 1). Importantly, it is worth noting that we did not observe any significant direct cytotoxicity for either Pep16 or levofloxacin (administered at 2 × MIC) on uninfected osteoblasts (MG-63) after 3 h of incubation (Figure S3).

**Figure 2.**
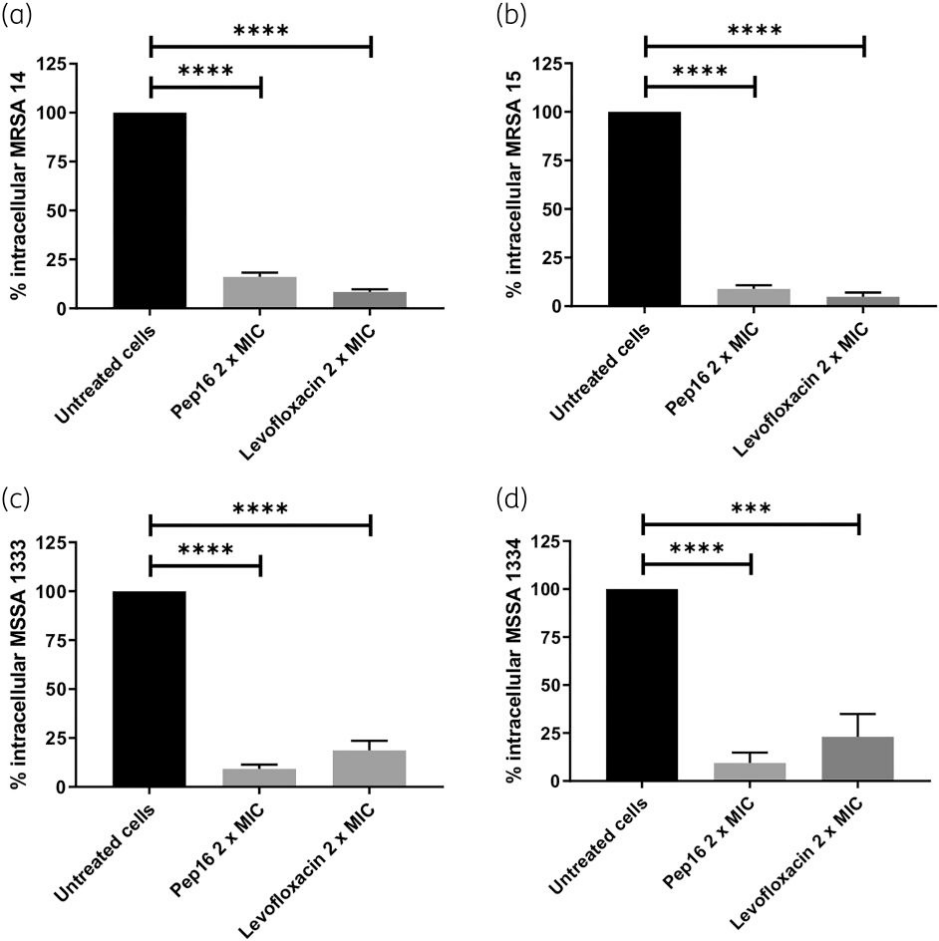
Intracellular activity of Pep16 and levofloxacin against intra-macrophagic *S. aureus* MRSA 1814 (a), MRSA 1815 (b), MSSA 1333 (c) and MSSA 1334 (d). At 3 h post-infection, TH-P1 cells were treated with Pep16 or levofloxacin at 2 × MIC for 24 h. Data represent the mean ± SD of three independent experiments. Intracellular cfu counts were normalized relative to untreated cells (depicted by black histograms), and differences between treated and untreated cells, as well as between the two compounds, were assessed using the Mann–Whitney *U*-test. ***P < 0.001; ****P < 0.0001.

In the acute model of macrophage infection, both Pep16 and levofloxacin (administered at 2 × MIC) were effective in significantly reducing the intracellular inoculum of the same four *S. aureus* clinical isolates (MRSA 1814, MRSA 1815, MSSA 1333 and MSSA 1334) 24 h after treatment, compared with untreated cells (Figure 2). Pep16 produced substantial reductions in the intracellular inoculum, with decreases of 83% (±1.8%), 91.9% (±2.2%), 90.1% (±2.6%) and 90.1% (±8.0%) for MRSA 1814, MRSA 1815, MSSA 1333 and MSSA 1334, respectively (Figure 2). No significant differences were found between levofloxacin and Pep16 in reducing intracellular MRSA and MSSA isolates (Figure 2). Similar to osteoblasts, no significant direct cytotoxicity was observed for either Pep16 or levofloxacin (at 2 × MIC) on uninfected macrophages (THP-1) after 24 h of incubation (Figure S3).

Altogether, we demonstrated that Pep16 exhibits substantial intracellular activity against both MRSA and MSSA strains in osteoblasts and macrophages, with comparable efficacy to levofloxacin, making it a promising candidate for the treatment of *S. aureus* septic arthritis.

### In vivo efficacy of Pep16 in a mouse model of septic arthritis

**Figure 3.**
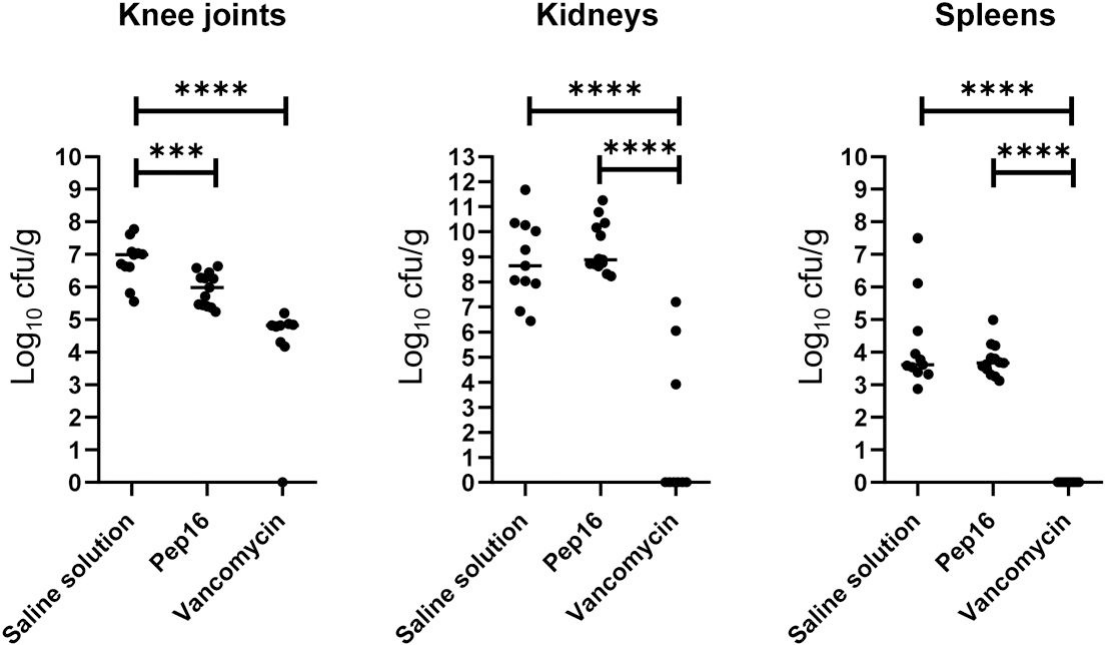
Bacterial loads (in log cfu/g) at Day 7 in knee joints, kidneys and spleen from mice infected by MSSA 1334. Mice were initially inoculated with approximately 1 × 10^8^ cfu of MSSA 1334. At 3 h post-infection, mice were treated with either with 10 mg/kg of Pep16 or 100 mg/kg of vancomycin, while untreated control mice received a saline solution without antibiotics. Treatments were administered via subcutaneous injection and continued daily until the conclusion of the experiment. Individual dots on the graph represent cfu counts for single animals (enumerated at Day 7), with black horizontal lines indicating median values. The experiments were performed with five mice per condition, in triplicate for Pep16 and saline solution, and in duplicate for vancomycin. Statistical analysis was conducted using the Mann–Whitney *U*-test. ***P < 0.001; ****P < 0.0001.

Based on these promising results, we proceeded to assess the efficacy of Pep16 in a mouse model using only one isolate (MSSA 1334) due to ethical reasons and since all four isolates had similar *in vitro* and intracellular activities. Note that all mice, untreated or treated by Pep16 or vancomycin, had survived at Day 7 (Figure S4). After subcutaneous treatment with Pep16 at a concentration of 10 mg/kg, a significant (∼10-fold) reduction in cfu counts was observed within the knee joints of infected mice 7 days after infection, in comparison with untreated mice. However, this reduction was not observed in kidneys or spleen (Figure 3). A similar reduction in cfu counts was noted in knee joints, kidneys and spleen of mice treated with vancomycin (at 100 mg/kg), compared with control mice (Figure 3).

**Figure 4.**
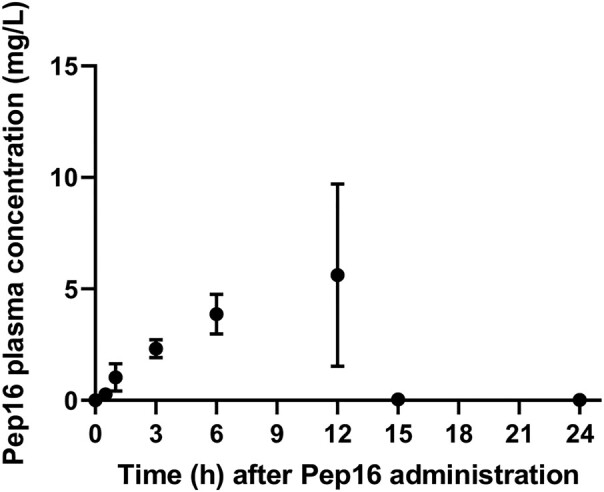
Plasma pharmacokinetics of Pep16 in mice after a single subcutaneous administration of 10 mg/kg. Dots from represent the mean ± SEM of three replicates.

Additionally, a pharmacokinetic analysis of Pep16 following a single subcutaneous administration of 10 mg/kg revealed a gradual increase in plasma concentrations over the course of 12 h, with a *C*_max_ of 5.6 mg/L (Figure 4).

Here, we demonstrate that Pep16, administered subcutaneously, significantly reduces load in the knee joints of infected mice, underscoring its potential as an effective treatment for *S. aureus* septic arthritis, while its peculiar pharmacokinetic profile needs further investigation.

## Discussion

The aim of this research was to explore Pep16’s antimicrobial potential against *S. aureus*, a key pathogen implicated in BJIs, by using a comprehensive investigation, designed to reflect clinical scenarios.

Notably, Pep16 exhibited a substantial reduction in the intracellular burden of *S. aureus* within both osteoblasts and macrophages, demonstrating comparable efficacy to the reference antibiotic, levofloxacin—a fluoroquinolone antibiotic known for its *in vitro* effectiveness against intracellular Gram-positive bacteria, including *S. aureus*.^22^ Importantly, no drug-induced cytotoxicity was observed at the tested concentrations. In comparison with newer contenders like dalbavancin and rifamycins, Pep16 showcased remarkable intracellular activity. Chauvelot *et al.*^11^ showed that dalbavancin, a recently introduced extended-duration lipoglycopeptide, significantly reduced the presence of *S. aureus* in osteoblasts starting at its MIC (0.125 mg/L), demonstrating a dose-dependent effectiveness. However, Pep16 outperformed dalbavancin, achieving reductions of 86.6% for MRSA isolates and approximately 63% for MSSA isolates. In contrast, the peak of reduction by dalbavancin at 100× its MIC was only 51.6%.^11^ In another study, conducted by Abad *et al.*^19^ on rifamycins, a group of antibiotics that inhibit bacterial RNA polymerase, the potential intracellular activity of these drugs against *S. aureus* was highlighted. When examining concentrations that were 10× or 100× their respective MICs, all rifamycins exhibited significant intracellular activity. However, the extent of reduction in intracellular bacteria varied: rifampicin reduced it by approximately 54.0%, rifapentine by 45.3%, and rifabutin stood out with a reduction of around 62.1%. The intracellular activity of Pep16 against *S. aureus*, compared with dalbavancin and rifamycins, further underscores its potential as an effective agent. Pep16’s unique structural composition, which includes non-natural amino acids,^18^ suggests enhanced cellular penetration, potentially supported by its amphiphilic properties. However, it is crucial to recognize that intracellular antimicrobial activity relies on factors beyond cellular concentration, including distribution within the intracellular compartment and the phagolysosomal acidic environment.^23^

The mouse model of septic arthritis further confirmed Pep16’s therapeutic potential, resulting in a 10-fold reduction in cfu counts within knee joints. These results build upon previous findings, indicating the promise of Pep16 as a therapeutic option.^18,21^ Indeed, the study by Nicolas *et al.* illustrated Pep16 significant efficacy in animal models of MRSA infections (sepsis and skin abscesses) and its ability to limit resistance emergence in MDR pathogens. This robust defence against MRSA infections corroborates our results and extends Pep16 applicability beyond septic arthritis to include potential treatments for severe sepsis.^17,18^ The findings of Chosidow *et al.* present another dimension of Pep16’s therapeutic promise. In their peritonitis mouse model, Pep16 alone was modest in its impact. However, in combination with colistin it showcased a dramatic reduction in bacterial counts, also staving off colistin-resistant mutants.^21^ This underscores the potential of leveraging combination therapies with Pep16 to achieve better clinical outcomes and manage drug resistance.

Nonetheless, our study has certain limitations, with one being the absence of standardized methodologies for assessing intracellular activity and *in vivo* efficacy. This poses a challenge when comparing results across different studies. Methodological discrepancies, such as treatment timing, can significantly impact findings. In particular, the majority of studies opt for immediate post-infection treatment while only a few consider delayed treatments, such as 12 h or 7 days post-infection. This methodological choice can affect the relevance of the findings, especially in chronic osteomyelitis contexts, where patients are often treated long after the infection onset.^24^ Moreover, a crucial distinction observed among existing studies is the cell model selected. Numerous studies utilize macrophages and cell lines, which exhibit contrasting behaviours to bone cells, particularly primary cells, in the context of *S. aureus* infection. Macrophages, being professional phagocytes, harbour substantially higher numbers of *S. aureus* compared with osteoblasts. Notably, primary osteoblasts, in comparison with other non-professional phagocytic cells, internalize fewer bacteria but present a higher rate of cell survival post-infection. To address this disparity, we employed both macrophages and osteoblasts to provide a comprehensive understanding of the infection dynamics of *S. aureus*. However, there are significant behavioural differences between osteoblastic cell lines, like MG-63 used in this study, and primary osteoblasts, with the latter showing reduced cell death upon infection and greater bacterial persistence in phagosomes.^25^ While primary cells often present more clinically pertinent results, cell lines offer advantages in terms of consistency and availability. This emphasizes the importance of selecting a suitable bone cell model in evaluating the efficacy of BJI treatments. Also, variability in treatment durations and timings, especially in *in vitro* models involving osteoblasts and macrophages, could influence outcomes.

Additionally, our *in vivo* model involved a single clinical isolate, highlighting the need for broader validation across diverse clinical isolates. Strain-dependent responses may introduce variability, emphasizing the importance of determining the MIC of Pep16 across a wider spectrum of *S. aureus* isolates. Regarding the pharmacokinetic analysis on mice, after administering Pep16 subcutaneously at a dose of 10 mg/kg in mice, there was a steady increase in Pep16 plasma concentration, with a peak at 12 h. Still, the pharmacokinetic profile did not attain concentrations surpassing the MICs required to inhibit the studied strains. This discrepancy might be attributed to factors like metabolism, distribution or the potential binding of the compound to other plasma proteins, which warrants further investigation to elucidate the exact mechanism. Given the inherent variability in *in vivo* responses, it is imperative to evaluate Pep16 against a broader collection of clinical isolates in mice. Even though MICs of Pep16 are relatively higher than those of established antibiotics like vancomycin or levofloxacin, it is interesting to note that intracellular activity and *in vivo* efficacy are similar.

In summary, our study demonstrates that Pep16 could be a promising therapeutic option for treating septic arthritis caused by *S. aureus*. Furthermore, its substantial intracellular activity in both non-professional and professional phagocytic cells could also be relevant for treating chronic infections caused by *S. aureus*.

## Supplementary Material

dlae025_Supplementary_Data

## Supplementary data

Figures S1–S4 are available as Supplementary data at *JAC-AMR* Online.
