# Supplementary material for: Intracellular activity and in vivo efficacy in a mouse model of septic arthritis of the novel pseudopeptide Pep16 against Staphylococcus aureus clinical isolates
Source: JAC Antimicrob Resist. 2024 Feb 26;6(1):dlae025. doi: 10.1093/jacamr/dlae025 (PMC10895697; doi:10.1093/jacamr/dlae025)
Supplement: dlae025_Supplementary_Data [file dlae025_supplementary_data.docx]

**Figure S1.** Time-kill curves for MSSA 1333 (A), MSSA 1334 (B), MRSA 1814 (C) and MRSA 1815 (D) in the presence of two concentrations above the MIC (80 mg/L and 160 mg/L) of Pep16. The data are presented as mean values ± standard deviations (SD).


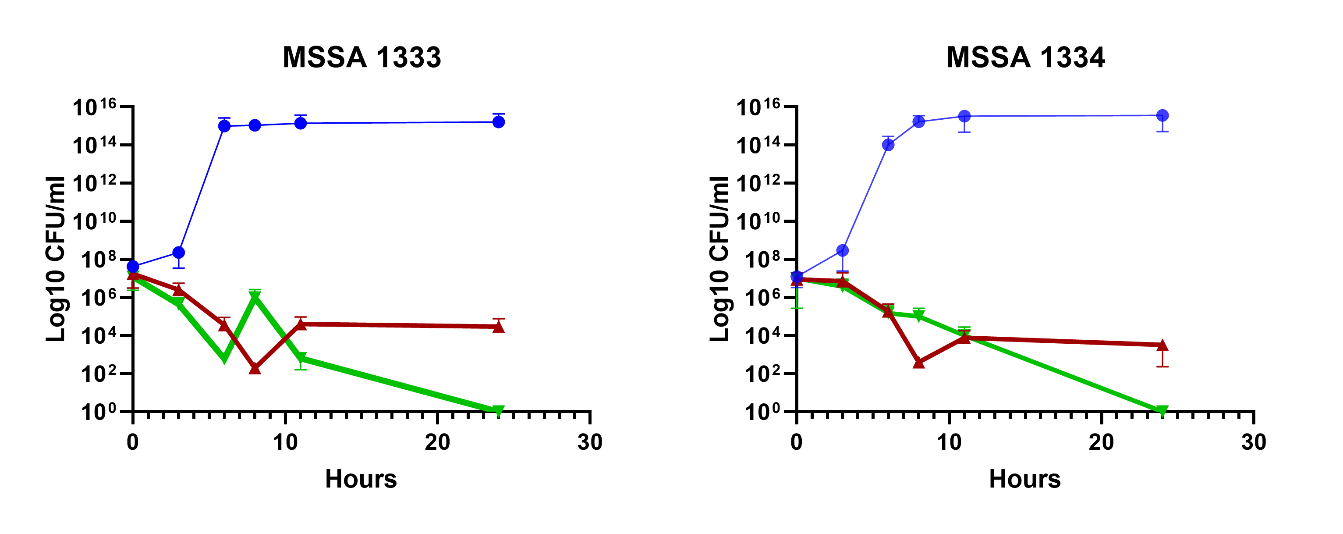

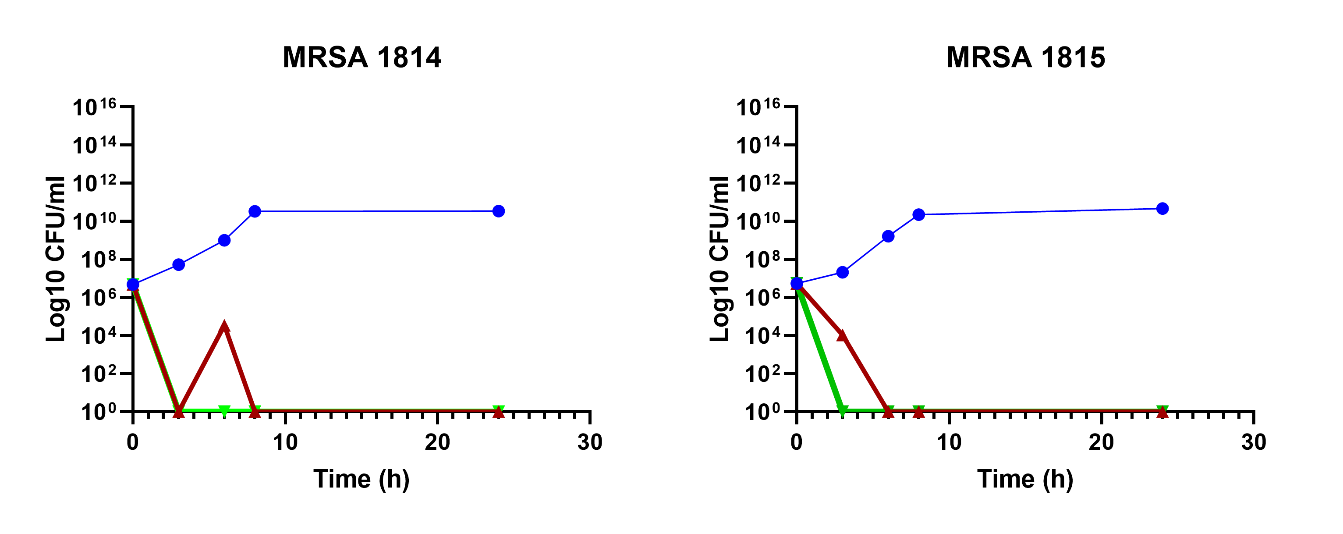


**A**

**B**

**C**

**D**


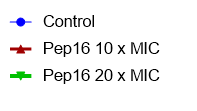


Log_10_ cfu/mL

Log_10_ cfu/mL

Log_10_ cfu/mL

Log_10_ cfu/mL

**Figure S2.** Summary of the fractional inhibitory concentration (FIC) of antimicrobial combinations against MRSA (1814 and 1815), MSSA (1333 and 1334) clinical isolates and the ATCC 29213 reference strain.

**_Pep16 + vancomycin_**

**Pep16 + teicoplanin**

**Pep16 + daptomycin**

**_Pep16 + l_evofloxacin**

**0.5**

**0.0**

**1.0**

**1.5**

**2.0**

**2.5**

**Fractional Inhibitory Concentration**

ATCC 29213

MRSA 1814

MRSA 1815

MSSA 1333

MSSA 1334

**Figure S3.** Viability of uninfected osteoblasts MG-63 (A) and macrophages THP-1 (B) after incubation with Pep16 or levofloxacin (at 2 x MIC). Osteoblasts were incubated 3 hours and macrophages 24 hours with antimicrobials.

**Untreated cells**

**Pep16 2 x MIC**

**Levofloxacin 2 x MIC**

**0**

**50**

**100**


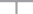

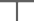


**% of viable osteoblasts**

**Untreated cells**

**Pep16 2 x MIC**

**Levofloxacin 2 x MIC**

**0**

**50**

**100**


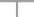

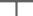


**% of viable macrophages**

**A**

**B**


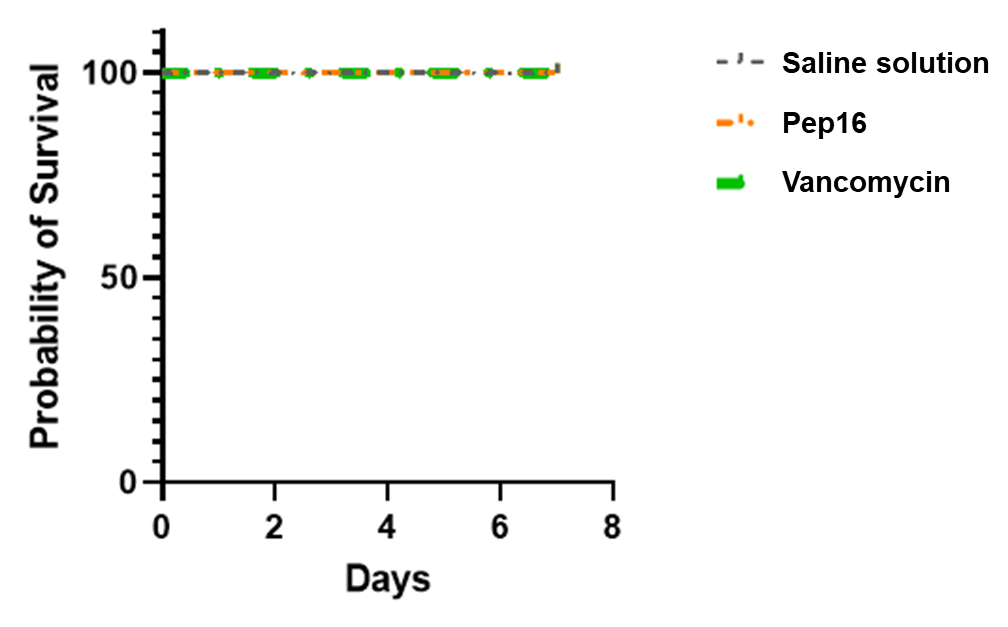


**Figure S4.** Survival of mice infected by MSSA 1334 and treated by Pep16 or vancomycin or not treated (saline solution).
